# Comparative Study of Two Table Grape Varieties with Contrasting Texture during Cold Storage

**DOI:** 10.3390/molecules20033667

**Published:** 2015-02-23

**Authors:** Troy Ejsmentewicz, Iván Balic, Dayan Sanhueza, Romina Barria, Claudio Meneses, Ariel Orellana, Humberto Prieto, Bruno G. Defilippi, Reinaldo Campos-Vargas

**Affiliations:** 1 Centro de Biotecnología Vegetal, Facultad Ciencias Biológicas, Universidad Andres Bello, República 217, Santiago, Chile; E-Mails: troyanomaster@gmail.com (T.E.); ivanbalicnor@gmail.com (I.B.); dayansanhueza@gmail.com (D.S); romibarria@gmail.com (R.B.); claudio.meneses@unab.cl (C.M.); aorellana@unab.cl (A.O.); 2 Instituto de Investigaciones Agropecuarias, INIA La Platina, Santa Rosa 11610, Santiago, Chile; E-Mails: hprieto@inia.cl (H.P.); bdefilip@inia.cl (B.G.D.)

**Keywords:** table grape, postharvest, berry texture, cell wall, calcium

## Abstract

Postharvest softening of grape berries is one of the main problems affecting grape quality during export. Cell wall disassembly, especially of pectin polysaccharides, has been commonly related to fruit softening, but its influence has been poorly studied in grapes during postharvest life. In order to better understand this process, the Thompson seedless (TS) variety, which has significantly decreased berry texture after prolonged cold storage, was compared to NN107, a new table grape variety with higher berry firmness. Biochemical analysis revealed a greater amount of calcium in the cell wall of the NN107 variety and less reduction of uronic acids than TS during cold storage. In addition, the activity of polygalacturonase was higher in TS than NN107 berries; meanwhile pectin methylesterase activity was similar in both varieties. Polysaccharide analysis using carbohydrate gel electrophoresis (PACE) suggests a differential pectin metabolism during prolonged cold storage. Results revealed lower pectin fragments in TS after 60 days of cold storage and shelf life (SL) compared to 30 days of cold storage and 30 + SL, while NN107 maintained the same fragment profile across all time points evaluated. Our results suggest that these important differences in cell wall metabolism during cold storage could be related to the differential berry firmness observed between these contrasting table grape varieties.

## 1. Introduction

The main challenge facing exporters of fresh fruit is the maintenance of postharvest quality. During transport, fruit undergoes modifications in organoleptic properties, thus affecting the perception of consumers. The table grape is not exempt from issues of degrading quality, and many problems have been detected during postharvest storage and shelf life (SL). Quality losses include weight loss, color change, berry softening and rachis browning, leading to reduced shelf life and overall quality [1]. The berry texture is one of the most important parameters that affects the table grape consumer [2]. Grape bunches with high berry firmness or a crunchy texture are considered a highly desirable attribute [3]. Despite the importance of this quality parameter, there is little information regarding the cellular factors that could be involved in fruit texture.

Softening during fruit ripening has been commonly related to changes of the structure and composition of pectin and hemicellulose polysaccharides of the cell wall and middle lamella [4]. Studies on grape also suggest a postharvest decrease of the pectin and hemicellulose fractions of the cell wall after cold storage [5]. Homogalacturonan (HG) has been proposed as one of the most important cell wall component involved in fruit texture changes [6,7]. This pectin polysaccharide is synthetized with a high degree of methyl-esterification and transported to the cell wall [8], where it is enzymatically processed during ripening by pectin methylesterase (PME; EC 3.1.1.11), leaving HG hydrolysable sites available for polygalacturonase (endo-PG; EC 3.2.1.15; and exo-PG; EC 3.2.1.67) and pectate lyase enzymes [6]. In addition, unesterified-HG negatively-charged sections generate calcium junctions between HG paired chains, thus contributing to increases in the strength of the cell wall [9].

It is known among growers that table grape varieties have differences in postharvest life span, which define the commercialization range of each one. Differences in berry texture among table grape varieties after long-term cold storage suggests that there are important genetic factors that define the characteristics of each variety and, thus, could influence berry texture. Our aim is to analyze the differences in cell wall metabolism during cold storage of two varieties contrasting in texture: Thompson seedless (TS), one of the most exported table grape varieties in Chile, and a new variety called NN107, which is known for its high firmness and crunchy texture.

## 2. Results and Discussion

### 2.1. Phenotypic Analysis of Grape Berries

Differences in berry firmness have been reported among several varieties of table grapes at harvest [2,10], which determine the firmness level during postharvest. In this study, the texture analysis showed significant differences between two table grapes varieties, where NN107 obtained significantly higher texture values associated with berry firmness at all times analyzed compared to Thompson seedless (TS) (Table 1). However, both varieties showed a decrease in firmness during postharvest, reaching a decline rate of 22.6% compared to harvest in the case of TS and 20.3% in NN107 at 60 + SL. Major differences between two varieties were registered after 60 days of cold storage. TS obtained a significant texture decline of 11.9% between 60 and 60 + SL points, while in NN107, this was only of 2.7% (Table 1). Quality losses of table grape during postharvest have been associated with shelf life after cold storage, which is a relevant factor of consumer rejection [11,12]. In this regard, NN107 showed less susceptibility to berry softening during shelf life compared to TS after long-term cold storage.

**Table 1:** Changes of berry texture (curve area) in Thompson seedless and the NN107 variety at harvest, after 30 and 60 days of storage at 0 °C and after two days of shelf life (SL) at 20 °C (30 + SL and 60 + SL, respectively). The rate of change relative to harvest is presented.

	Thompson Seedless		NN107	
Storage Time (days)	Curve Area (N·mm)	Rate Relative to Harvest (%)	Curve Area (N·mm)	Rate Relative to Harvest (%)
0 (harvest)	84 ± 10*a*	100	182 ± 38*a* *	100
30	84 ± 13*a*	100 (0)	166 ± 22*ab* *	91.2 (8.8)
30 + SL	80 ± 12*ab*	95.2 (4.8)	158 ± 17*bc* *	86.8 (4.4)
60	75 ± 10*b*	89.3 (5.9)	150 ± 19*bc* *	82.4 (4.4)
60 + SL	65 ± 11*c*	77.4 (11.9)	145 ± 25*c* *	79.7 (2.7)

Notes: Letters represent statistically-significant differences during postharvest storage (Tukey’s test, *p* < 0.05). * Significant differences between varieties at the same postharvest stage (Student’s *t*-test, *p* < 0.05). Values in brackets represent the rate of change relative to the previous data sample point.

In relation to other quality parameters, TS registered a significant diameter decline (5.3%) at 30 + SL compared to 30 days of cold storage (Table 2). The same tendency of decline was observed between 60 and 60 + SL points (3.2%). On the contrary, soluble solids content registered an increase in all evaluated postharvest moments compared to harvest, especially after shelf life (9.4% and 8.3% at 30 + SL and 60 + SL, respectively). Acidity showed a decrease of 0.1 g·L^−1^ (25%) in all evaluated postharvest moments compared to harvest, except at 60 + SL (Table 2). NN107 showed a similar trend as observed in TS, but some differences were observed. No significant decrease was found of the berry diameter associated with shelf life after 30 days (2.4%) or 60 days of cold storage (1.4%) (Table 3). Soluble solids registered a significant increase only after 60 + SL compared to harvest (7.7%), and acidity remained constant in all evaluated moments (Table 3). Such a diameter decline with a concomitant increase of soluble solids could be possibly associated with berry dehydration during postharvest storage. On this note, TS showed an earlier effect compared to NN107 during postharvest storage, specially associated with shelf life. These results, together with the texture data, indicate NN107 to be in a superior range compared to TS in terms of maintaining quality parameters during postharvest life, providing a suitable model for comparative biochemical studies.

**Table 2:** Quality parameters of Thompson seedless at harvest, after 30 and 60 days of storage at 0 °C and after two days of shelf life at 20 °C (30 + SL and 60 + SL, respectively). The rate of change relative to harvest is presented.

	Thompson Seedless					
	Berry diameter		Soluble solids content		Titratable acidity	
Storage Time (days)	Mean Value (mm)	Rate Relative to Harvest (%)	Mean Value (%w/w)	Rate Relative to Harvest (%)	Mean Value (g·L^−1^)	Rate Relative to Harvest (%)
0 (harvest)	19.0 ± 1.2*ab*	100	19.2 ± 1.6*c*	100	0.4 ± 0.0*a*	100
30	19.2 ± 1.3*a*	101.1 (−1.1)	20.3 ± 1.0*ab*	105.7 (−5.7)	0.3 ± 0.0*b*	75 (25)
30 + SL	18.2 ± 1.1*b*	95.8 (5.3)	21.0 ± 0.8*a*	109.4 (−3.7)	0.3 ± 0.0*b*	75 (0)
60	19.2 ± 1.0*a*	101.1 (−5.3)	20.1 ± 1.0*b*	104.7 (4.7)	0.3 ± 0.0*b*	75 (0)
60 + SL	18.6 ± 1.0*ab*	97.9 (3.2)	20.8 ± 1.1*ab*	108.3 (−3.6)	0.4 ± 0.0*a*	100 (−25)

Notes: Letters represent statistically-significant differences during postharvest storage (Tukey’s test, *p* < 0.05). Values in brackets represent the rate of change relative to the previous data sample point.

**Table 3:** Quality parameters of NN107 at harvest, after 30 and 60 days of storage at 0 °C and after two days of shelf life at 20 °C (30 + SL and 60 + SL, respectively). The rate of change relative to harvest is presented.

	NN107					
	Berry Diameter		Soluble Solids Content		Titratable Acidity	
Storage Time (days)	Mean Value (mm)	Rate Relative to Harvest (%)	Mean Value (%w/w)	Rate Relative to Harvest (%)	Mean Value (g·L^−1^)	Rate Relative to Harvest (%)
0 (harvest)	20.7 ± 1.5*a*	100	18.2 ± 0.9*b*	100	0.4 ± 0.0*a*	100
30	20.3 ± 1.1*ab*	98.1 (1.9)	18.1 ± 0.6*b*	99.5 (0.5)	0.4 ± 0.0*a*	100 (0)
30 + SL	19.8 ± 0.8*b*	95.7 (2.4)	18.6 ± 0.8*b*	102.2 (−2.7)	0.4 ± 0.0*a*	100 (0)
60	20.0 ± 0.9*ab*	96.6 (−0.9)	18.7 ± 0.8*b*	102.7 (−0.5)	0.4 ± 0.0*a*	100 (0)
60 + SL	19.7 ± 1.3*b*	95.2 (1.4)	19.6 ± 1.1*a*	107.7 (−5.0)	0.4 ± 0.0*a*	100 (0)

Notes: Letters represent statistically-significant differences during postharvest storage (Tukey’s test, *p* < 0.05). Values in brackets represent the rate of change relative to the previous data sample point.

### 2.2. Calcium Content

**Figure 1:**
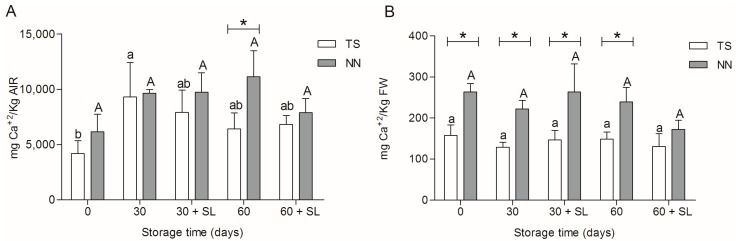
(**A**) Calcium content expressed in mg per kg of alcohol insoluble residues (AIR); (**B**) calcium content expressed in kg of fresh weight (FW). Analysis was carried out during 30 and 60 days of storage at 0 °C and after two days of shelf life at 20 °C (30 + SL and 60 + SL, respectively). Calcium was measured on Thompson seedless (TS, white bar) and NN107 (NN, gray bar) by inductively-coupled plasma-optical emission spectroscopy (ICP-OES). Letters represent statistically significant differences in TS (lower case) and NN (upper case) during postharvest storage (Tukey’s test, *p* < 0.05). * Significant differences between varieties at the same developmental stage (Student’s *t*-test, *p* < 0.05).

Calcium has been commonly associated with fruit texture due to its structural role in the cell wall through the formation of ionic bridges between homogalacturonan (HG) chains, according to the model known as “egg boxes” [13]. In order to explore putative differences in structural calcium between TS and NN107 grape berries, we measured calcium levels in cell wall material obtained by alcohol insoluble residues (AIR) and from whole fresh grape berries obtained during different stages of postharvest storage (Figure 1).

Results of calcium determination in cell wall material showed a significant increase in calcium after 30 days of cold storage in TS grape berry samples, while NN107 showed the same level of calcium across all postharvest time points (Figure 1A). Differences between both varieties were observed only after 60 days of cold storage, where AIR samples of NN107 revealed significantly higher levels of calcium compared to TS. Results suggest few changes in calcium binding sites (pectins) in the firmer variety, while TS showed first a significant increase followed by a decrease during cold storage. Since calcium quantification was performed from the same amount of AIR in all determinations, these calcium levels increased in TS could be interpreted as an enrichment of the pectin/calcium proportion in AIR due to higher depolymerization of pectin not bound to calcium in the TS cell wall compared to NN107. Thus, determination of cell wall metabolism between NN107 and TS could be an interesting inquiry to pursue.

Additionally, determinations based on fresh weight berries showed significantly higher amounts of calcium in NN107 samples in all evaluated postharvest moments, except 60 + SL, compared to TS berry samples (Figure 1B). Since calcium plays an essential role in cellular functions, plant cells have different mechanism for calcium storage in organelles to ensure proper homeostasis [14,15]. Higher amounts of calcium in fresh weight samples of the firmer variety could suggest a differential calcium homeostasis response, which could indicate a higher calcium demand in NN107 compared to TS. 

### 2.3. Uronic Acid Determination

**Figure 2:**
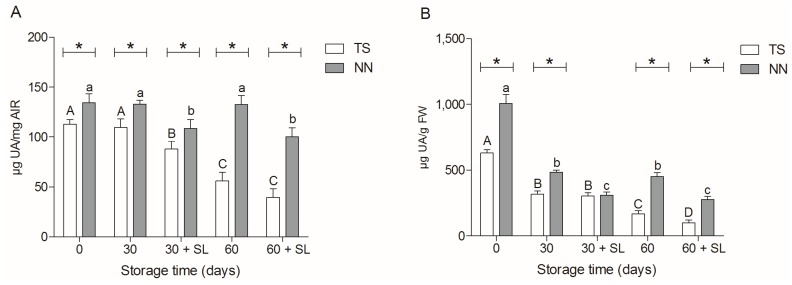
(**A**) Uronic acid (UA) content expressed in mg of AIR; (**B**) UA content expressed in µg per g of FW. Analysis was carried out during 30 and 60 days of storage at 0 °C and after two days of shelf life at 20 °C (30 + SL and 60 + SL, respectively). UA analysis was performed on the Thompson seedless (TS, white bar) and NN107 (NN, gray bar) variety. Letters represent statistically-significant differences in TS (lower case) and NN (upper case) during postharvest storage (Tukey’s test, *p* < 0.05). * Differences between varieties at the same evaluation moment (Student’s *t*-test, *p* < 0.05).

In order to identify characteristics associated with the berries texture, we determined the uronic acid in the cell wall of the TS and NN107 variety during postharvest storage. Results expressed on the basis of AIR suggested a decrease in the uronic acid fraction in total cell wall material in both varieties after harvest (Figure 2A). However the uronic acid in AIR was significantly higher in the NN107 variety in almost all evaluated moments, except 30 + SL. Results expressed on the basis of fresh weight berry revealed that TS has a reduction of about 50% of the uronic acid after 60 days of storage and 60 + SL compared with the moment of harvest (Figure 2B). On the contrary, NN107 showed a significant decrease of uronic acid only after shelf life. Comparison between two varieties suggests that NN107 has greater amounts of uronic acid than TS at all times during postharvest storage, which correlates with the texture data. These results suggest a differential metabolism of uronic acid between varieties.

### 2.4. Activity of Cell Wall Modifying Enzymes

**Figure 3:**
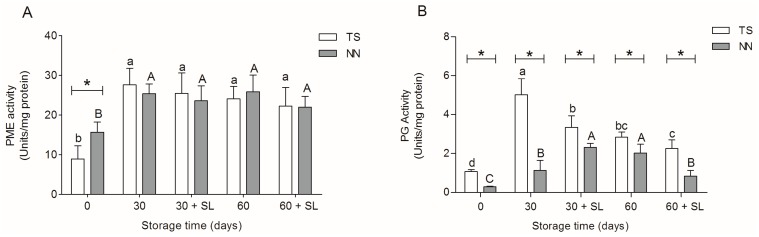
(**A**) Pectin methylesterase (PME) activity and (**B**) polygalacturonase (PG) activity of grape berries of the Thompson seedless (TS, white bars) and NN107 (NN, gray bars) varieties. Samples were obtained from grape bunch storage during 30 and 60 days at 0 °C, and after shelf life at 20 °C post-cold storage (30 + SL and 60 + SL, respectively). Letters represent statistically-significant differences in TS (lower case) and NN (upper case) during postharvest storage (Tukey’s test *p* < 0.05). * Statistically significant differences between both varieties for each evaluation moment (Student’s *t*-test, *p* < 0.05).

Postharvest softening has been generally correlated with the activity of several cell wall-degrading enzymes [6]. Analysis of the activity of pectin methylesterase (PME) and polygalacturonase (PG) was carried out for all postharvest storage time points. PME activity showed similar patterns in both varieties, with a significantly increase after 30 days of cold storage, as compared with harvest; after this point, it was maintained during all storage times (Figure 3A). Despite the fact that PME activity has no correlation with the postharvest softening of the grapes in the current analysis, this enzyme could be involved with other cell wall modifications, such as a decreasing methyl esterification, which contributes to increases in the substrate availability to others cell wall hydrolases [16]. Additionally, PME activity could affect the hydration and solubilization of pectins, pH change and modification of the apoplast ionic balance [6]. Thus, the PME activity with other remodeling cell wall enzymes has been associated with postharvest dehydration of grape berries, suggesting an important role in cell wall disassembly during weight loss [17,18]. Furthermore, this could be correlated with the reduction of berry diameter and the accumulation of soluble solids (Table 2 and Table 3).

In the case of PG activity (Figure 3B), TS showed a significant increase in activity after 30 days of cold storage, followed by a decrease throughout the rest of postharvest storage. NN107 also showed an increase in PG activity, raising its peak at 30 + SL, followed by a gradual decrease. Comparing the PG activity between both varieties, it was observed that NN107 presented a significantly lower amount than TS in all postharvest time points (Figure 3B). Since the activity of this enzyme is closely related to the softening of the fruit, there is evidence that activity correlates with HG depolymerization and solubility of pectin polysaccharides [6,9]. Our previous published results suggest that the PG activity of TS increases throughout maturation, paralleling reported trends in others grape varieties [19]. However, the higher PG activity in TS at 30 days was five-fold of NN107 with a concomitant decrease of the uronic acids (Figure 2). Interestingly, PG showed a high activity at 0 °C in TS, suggesting a higher susceptibility of TS to having soft berries after prolonged cold storage. It is known that PG has a low activity during ripening, which increases after harvest in grapes [17,19]. Similarly, in apples and pears, PG activity has been reported to increase during cold storage and correlates with cell wall degradation [20,21]. In the case of the firmer NN107 variety, our previously reported results showed a decrease of PG activity during ripening of grape berries, while uronic acids remain constant, in an opposite way from TS [19]. Postharvest results in the NN107 variety revealed a lower increase in PG activity compared with TS, reaching a maximum activity of three-fold higher than harvest values, correlating with a lower decline in uronic acids level (Figure 2A). Such differences in PG activity could be a biochemical indicator of more complex metabolic differences between both varieties involving differential cell wall-degrading activities.

### 2.5. Carbohydrate Electrophoresis

**Figure 4:**
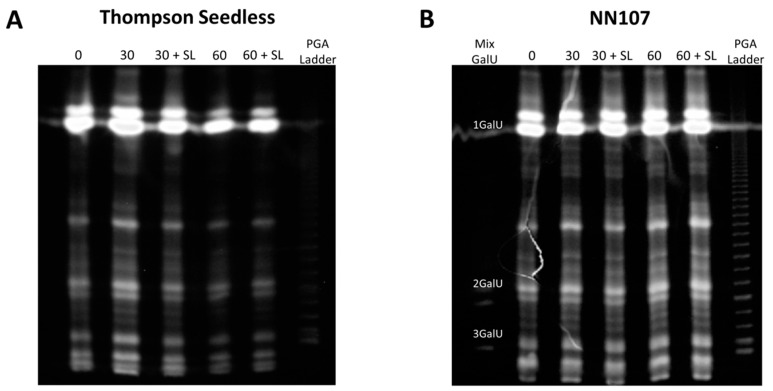
(**A**) Polysaccharide analysis using carbohydrate gel electrophoresis (PACE) of the AIR samples of the Thompson seedless and (**B**) NN107 grape variety. Fruit samples were obtained after 30 and 60 days of storage at 0 °C and after two days of shelf life at 20 °C (30 + SL and 60 + SL, respectively). AIR was hydrolyzed with a commercial pectolyase. PGA ladder corresponds to the oligo galacturonic acid ladder. Mix GalU corresponds to a mixture of monomers, dimers and trimers (GalU, 2 GalU and 3 GalU, respectively).

Polysaccharide analysis using carbohydrate gel electrophoresis (PACE) was performed in order to analyze pectin fragments of AIR samples of both varieties obtained by enzymatic hydrolysis with a pectolyase and derivatized with 2-amino-acridone (AMAC) fluorophore. No differences were observed in the patterns of electrophoretic bands between different postharvest samples in the two grape varieties (Figure 4). However, samples of TS showed differences in the intensity of all bands after 60 days of cold storage and 60 + SL (Figure 4A). Since the same amount of digested cell wall material was loaded in each lane, these results could suggest a reduction in the pectin proportion of AIR in those samples, in agreement with the reduction observed in uronic acid content in the same evaluated moments of TS samples (Figure 2). On the other hand, samples of NN107 showed both the same patterns and intensity of oligosaccharide fragments in all postharvest evaluated moments (Figure 4B). This information correlated with the activity of cell wall-degrading enzymes shown in Figure 3.

Taken together, these results suggest differences in cell wall metabolism that could strongly influence the texture contrast observed between TS and NN107 during harvest and postharvest. Thus, the firmer variety showed a lower PG activity accompanied by higher calcium availability. On the other hand, TS presented higher PG activity and lower calcium availability. Such a relationship between PG and calcium suggest higher pectin integrity in the NN107 berries compared to TS, in agreement with previously-reported evidence that suggests a competitive inhibitor role for calcium in PG activity [22,23]. In addition, higher levels of uronic acids and more stable oligosaccharide band patterning in PACE experiments associated with the firmer variety are in agreement with the proposed involvement of cell wall integrity in grape berry texture.

## 3. Experimental Section

### 3.1. Plant Material and Maturity Parameters

*Vitis vinifera* cv. TS and cv. NN107 grape bunches were collected at a commercial vineyard located in Llay-Llay (Valparaíso, Chile) during the 2011–2012 season between December to March. Grape bunches were harvested at full maturity stages according to commercial parameters for each variety (Table 2 and Table 3). After harvesting, the grapes were packaged conventionally according to industry practices and were stored in cold conditions (0 °C) for 30 to 60 days. After each cold period, grapes were exposed to 20 °C for two days (shelf life, SL). The diameter, texture, soluble solids and acidity were determined after harvest, cold storage (30 and 60 days) and shelf life (30 + SL and 60 + SL). The diameters (mm) of 25 grape berries belonging to five different grape bunches were measured with a caliper. Soluble solids content (%w/w of g sucrose per 100 g solution) of the same fruit was measured with a temperature-compensating digital refractometer (HI 96811, Hanna Instruments Inc., Woonsocket, RI, USA). Titratable acidity (g·L^−1^ of tartaric acid) of three pooled juices extracted from the same berries was measured by titration with 0.1 N NaOH to an end point (pH 8.2). Additionally, a fraction of each grape bunch was frozen in liquid nitrogen and stored at −80 °C for biochemical analysis. 

### 3.2. Texture Analysis

In order to approach a representative value of crunchy texture, we performed a penetration test of whole grape berry adding the skin contribution in the texture analysis. For that, we used a TA-XT plus Texture Analyzer (Stable Micro Systems Ltd., Surrey, UK) equipped with the Volodkevich Bite Jaw (VB) probe, which simulates an incisor tooth, following similar parameters of texture analysis utilized in other fruits [24,25,26,27]. The texture of the same group of 25 grape berries was evaluated through the entire fruit along the middle of the equatorial plane of the berry using the VB probe. Complete force-time curves were obtained, penetrating 15 mm, with a test speed of 1 mm·s^−1^, and the areas under the curves (N·mm) were calculated by the Exponent Lite software provided with the texturometer (Exponent Lite, version 5.0.9.0; Stable Micro Systems Ltd., Surrey, UK).

### 3.3. Alcohol-Insoluble Residue Preparation

Cell wall material was obtained by the alcohol-insoluble residue method based on Lefever [28] with modifications. Grapes berries (10 g) were ground with a mortar and pestle in 50 mL of ethanol 80%. Then, 50 mL of 80% ethanol were added, and this was boiled for 20 min at 80 °C. The mixture was cooled at room temperature (RT). The solid residue was filtered over Miracloth (CalBiochem, La Jolla, CA, USA) and was resuspended and stirred for 30 min in 25 mL ethanol 80% and filtered again. This procedure was performed 3 times. The solid residue was resuspended in 25 mL ethanol 95%, filtered and solubilized in 25 mL acetone 100% and filtered again. The final AIR was dried overnight at RT.

### 3.4. Uronic Acid Analysis

AIR samples (2 mg) were hydrolyzed with 0.25 mL of trifluoroacetic acid 2 M (Merck, Darmstadt, Germany) for 3 h at 100 °C. Then, the acid was evaporated, and the samples were washed three times with 100% isopropanol. The cell wall material was suspended in 0.2 mL ultrapure water and sonicated for 10 min. The suspension was centrifuged at 9,000× *g* for 5 min, and the supernatant was used for analysis. The uronic acid content was determined by the method described by Blumenkrantz and Asboe-Hansen [29] with modifications. To the reaction mixture was supplemented 0.01 mL of sulfamic acid 4 M (pH 1.6) to eliminate the interference by neutral sugars.

### 3.5. Polysaccharide Analysis Using Carbohydrate Gel Electrophoresis

AIR samples were suspended in water to a final concentration of 0.5 mg·mL^−1^, were mechanically homogenated with a tissue grinder and dried in a vacuum concentrator (Concentrator plus, Eppendorf, Hamburg, Germany). AIR was digested with 3 µL of pectolyase (P3026, Sigma, Dorset, UK) prepared to final concentration of 10 mg·mL^−1^ in 250 µL of sodium citrate buffer 0.01 M (pH 4.8) for 1.5 h at 38 °C and then heated to 100 °C for enzymatic inactivation. Derivatization with 2-amino-acridone (AMAC) was performed according to Goubet [30]. Polyacrylamide electrophoresis was performed at 12 °C in 25% (w/v) acrylamide-bis-acrylamide in the resolving gel and stacking gel of 10% (w/v) acrylamide-bis-acrylamide. A discontinuous electrophoresis buffer system was used using 0.15 M Tris-glycine pH 8.5 as the cathode and 0.1 M Tris-Cl pH 8.2 as the anode. The samples were electrophoresed initially at 200 V for 20 min and then at 1,000 V for 3 h. Each gel was loaded with two types of standards: a commercial mix of galacturonic acid monomers (GalA), dimmers (GalA)2 and trimers (GalA)3 (Sigma-Aldrich, St. Louis, MO, USA). The other standards correspond to different oligogalacturonic acids (OGAs) obtained via autoclaving of polygalacturonic acid in a water solution (0.5 mg·mL^−1^) for 20 min at 121 °C, according to Goubet [30]. The gel image was obtained using a UV transilluminator (ECX-F20.M, Vilber Lourmat, Marine la Valeé, France) coupled with a CCD camera (C-5060, Olympus, Tokyo, Japan) and handled with Doc-It LS image analysis software (UVP Inc., Upland, CA, USA).

### 3.6. Calcium Quantification

The calcium content of the AIR and the fresh weight of the total grape was determined using three biological replicates by inductively-coupled plasma optical emission spectrometry (ICP-OES), using a iCAP 6000 Series ICP Emission Spectrometer (Thermo Scientific, Cambridge, UK). Fifty milligrams of each sample were digested in 10 mL of 65% ultrapure nitric acid and 37% ultrapure chloride acid solution. The settings of the operations were as follows: RF power, 1.15 kW; nebulizer flow, 0.7 L·min^−1^; auxiliary gas flow, 0.5 L·min^−1^; plasma gas flow, 13.2 L·min^−1^; analysis pump rate, 50 rotations·min^−1^; sample delay time, 0 s; and sample flush time, 30 s. Samples were measured at 393.3 nm. The control elemental stock standards were purchased from Merck. Results are expressed in parts per million (ppm) according to a prior calibration curve.

### 3.7. Protein Extraction

Proteins were extracted according to the method described by Deytieux-Belleau [31]. Grape berries (5 g) were ground to a fine powder in liquid nitrogen. Protein extraction was performed at 4 °C in 5 mL of solution containing 0.1 M Tris-HCl buffer, pH 7, 13 mM EDTA, 20 mM β-mercaptoethanol, 1 M NaCl, 1% polyvinylpyrrolidone (w/v), 20% glycerol (v/v) and 1% Triton X-100. The mixture was centrifuged at 9,000× *g* for 20 min. The supernatant was extracted with five volumes of chloroform/methanol (1:4) and three volumes of distilled water and then centrifuged at 9,000× *g* for 10 min. The supernatant was removed (without removing the interface) and extracted with three volumes of methanol; then, the supernatant was discarded and the pellet suspended in 5 mL of Tris-HCl buffer 0.1 M (pH 7.5) to solubilize the crude extract. The protein content was determined using the Bradford method [32] with bovine albumin serum as the standard.

### 3.8. Pectin Methylesterase Activity

The PME activity was measured as described by Hagerman and Austin [33] with modifications. The reaction mixture contained 1 mL of pectin solution composed of 0.5% w/v of pectin from citrus peel (Sigma-Aldrich, St. Louis, MO, USA) in water adjusted to pH 7.5 with NaOH, 0.075 mL bromothymol blue solution (0.01% w/v in 3 mM phosphate potassium buffer, pH 7.5) and 0.425 mL protein extract. The absorbance was measured immediately at 620 nm and after 30 s. PME activity was determined using a standard curve described by Hagerman and Austin [33]. One unit (U) is defined as the amount of the enzyme required to produce 1 µmol of GalA per second. PME activity was performed in triplicate using three different protein extractions from different grape bunches.

### 3.9. Polygalacturonase Activity

The PG activity was measured according to the method described by Lohani [34]. The reaction mixture contained 0.3 mL of 200 mM buffer sodium acetate (pH 4.5), 200 mM NaCl, 0.3 mL of polygalacturonic acid (PGA 1% w/v, Sigma-Aldrich) and 0.1 mL of protein extract. The reaction mixture was incubated for 15 min at 37 °C and terminated via heating for 5 min. The enzymatic activity was determined by the colorimetric assay using 100 µL of 3,5-dinitrosalicylic acid (Sigma-Aldrich) with 100 µL of reaction mixture and heating for 15 min at 100 °C. The formation of reducing groups was estimated against a standard curve of D-glucose (USBiologicals, Swampscott, MA, USA) measured at 540 nm. One unit (U) of enzyme was defined as the amount of enzyme required to liberate 1 µg·mL^−1^ of D-glucose per min. PG activity was performed in triplicate using three different protein extractions from different grape bunches.

### 3.10. Statistical Analysis

Differences between the analyzed parameters were statistically evaluated using an analysis of variance (ANOVA), and the mean comparisons between varieties were determined using Student’s *t*-test at *p* < 0.05 using the R statistical analysis package version 3.0.1. (R Foundation for Statistical Computing, Vienna, Austria) [35]. The mean comparisons across different postharvest conditions for each variety were determined using Tukey’s test at *p* < 0.05.

## 4. Conclusions

Our results suggest an important association of pectin metabolism in the cell wall of grape berry, with differences in observed texture between Thompson seedless and NN107 at harvest and during cold storage and shelf life. High calcium content, low uronic acid degradation and reduced PG activity could be associated with a firmer grape berry texture phenotype and higher fruit quality for prolonged cold storage. These results confirm the biochemical and physiological differences previously published in our pre-harvest studies [19]. Thus, these varieties represent a great model of berry texture studies. More comprehensive transcriptome or proteomics studies are needed to obtain deeper insights into this and other quality parameters and to open the possibility of finding relevant biomarkers for grape breeding programs.
